# Women outweighed men at life expectancy in Bangladesh: does it mean a better quality of life?

**DOI:** 10.1016/j.heliyon.2021.e07618

**Published:** 2021-07-16

**Authors:** Md. Zakiul Alam

**Affiliations:** Department of Population Sciences, University of Dhaka, Dhaka 1000, Bangladesh

**Keywords:** Life expectancy, Multimorbidity, Healthy life expectancy, Loneliness, Depression, Active ageing, Quality of life, Failures of success, Bangladesh

## Abstract

**Introduction:**

Men had a higher life expectancy than women until 2000 in Bangladesh. After 2000, statistics showed that women had a higher life expectancy than men. We aimed to address whether higher life expectancy is a gain or burden (Failures of Success) for older women.

**Methods:**

We utilised data from *Bangladesh Demographic and Health Survey-2011*, *Health and Morbidity Status Survey-2014, Population and Housing Census-2011, Household Income and Expenditure Survey-2010,* and primary data. We calculated and estimated gender-specific different aspects of quality of life.

**Findings:**

Findings showed that the mean age at marriage was 5.8 years lower for women, while life expectancy was three years higher than men; thus, women were expected to live for 8.8 years alone. Both disabilities and morbidities were higher among women than men; hence they had lower healthy life expectancy. About 53% of women could work daily activities while 8% higher for men. Only 2.4% of women earned while almost 58% for men. Depression and loneliness were also higher among women than men. The value of the active ageing index and quality of life were also lower for women than men.

**Conclusion:**

This study overall found that there were the ***Failures of Success*** especially for older women in Bangladesh, as increasing life expectancies led them to extra years of chronic illness, economic insolvency, more anxiety and depression, and increasing misery. Therefore, sex and gender and their inherent differences should inform decision making to promote gender equity in health. The government and policymakers may intervene for quality of life, especially for women, through reducing gender stereotypes and increasing community engagement. Without considering the quality of life, healthy ageing cannot be ensured.

## 1 Introduction

Life expectancy is the average number of years a person born is expected to live if age-specific mortality rates remain constant in the future [1] and considered one of the three indicators of human development [2]. Due to the changes in disease patterns from communicable to non-communicable, there is an advancement in life expectancy [3]. Globally, there has been substantial improvement in average life expectancy from 47 years in 1950 to 70.8 years in 2015 and is anticipated to 82.4 years by 2100 [4]. Bangladesh, like other countries, has also experienced a mortality transition with the increase of life expectancy. In 1990, life expectancy at birth in Bangladesh was 58 years, with the under-five mortality of 144 per 1000 live births and maternal mortality of 590 per 100,000 live births [5]. With reduced under-five mortality to 31 and maternal mortality to 172 in 2017, life expectancy at birth has increased to 72 years [6].

Women have 3–6 years of higher life expectancy than men, irrespective of whether they come from developed or developing countries [4, 7]. In Bangladesh, men had a higher life expectancy at birth than women until 2000 [5,6] due to gender disparities and solid patriarchal norms. After 2000, women outweighed men in life expectancy with their biological superiority [7, 8] and substantial reduction in mortality [7, 9, 10]. However, gains in life expectancy do not necessarily mean a better quality of life; instead, disability-free and healthy life is essential as during the measurement of the burden of diseases, morbid or disable state is often considered dead since the duration of diseased or disability is excluded from the total life expectancy [11].

The loss of spouse, or the family and social level isolation especially at the older age cause loneliness. Living a lonely life obstructs the quality of life, as loneliness may sometimes results in anxiety and depression. Moreover, the increasing number of the older population, which results from a gain in life expectancy, is often economically vulnerable and dependent on others. Active ageing and involvement in economic activities may reduce vulnerability. However, around the world, most people retire after the age of 60 or 65 years [12], making them more alienated and dependent after this period if they do not have more income generating activities [13].

Though women live longer, in most situations, they have more morbidity, disability, depression and loneliness [7, 14], which is often labelled as paradox [7]. This paradox (new paradigm) is described as the ***Failures of Success*** [15], which asserts that increasing life expectancy would lead to additional years of chronic illness, economic hardship, and increasing suffering for many older adults. In contrast, the ***Compression of Morbidity*** hypothesis argues that the age of onset of chronic diseases may be postponed more than the age at deceased, squeezing many morbidities in life into a shorter period with less lifetime disability [16]. Nevertheless, empirical findings do not support the existence of compression of morbidity when it is defined as major diseases and mobility functioning loss [17].

Changing patterns of life expectancy is tracking all over the world as a part of vital registration system, which is also found at world development indicators [5]. Globally, as well as in Bangladesh, existing studies have focused primarily on overall life expectancy, disability-free, or healthy life expectancy, but hardly any focus have been provided on the broad aspects of quality of life [18, 19, 20, 21, 22, 23]. The Bangladesh Bureau of Statistics (BBS) has been publishing vital statistics since 1980. That only includes life expectancy for men and women but excludes healthy life expectancy or quality of life [6, 10, 24]. Moreover, it is also presumed that women may enjoy less quality of life than men due to a patriarchal and male-dominated society [25, 26] and constraint choice [14], and reduced individual agency [7]. In this regard, we aimed to describe the different aspects of quality of life for older men and women to address whether the higher life expectancy at birth is a gain or burden (***Failures of Success***) for older women in Bangladesh. This study will help Bangladesh design specific policies to improve the quality of life for older adults, especially for older women. Consequently, it will help to enjoy a better life especially by older women and to achieve health-related goals of sustainable development.

## 2 Methodology

### 2.1 Data sources

This study utilised both secondary and primary sources of data to address broad aspects of the quality of life of the older population. Data were taken from *Bangladesh Demographic and Health Survey (BDHS) 2011* [25], *Health and Morbidity Status Survey (HMSS) 2014* [27], *Population and Housing Census 2011* [28,29] and *Household Income and Expenditure Survey (HIES) 2010* [30]. Since there were multiple data sources, we took the census and surveys from a similar time frame (between 2010 and 2014).

#### 2.1.1 Primary data collection

This study used a cross-sectional research strategy for collecting primary data. Primary data were collected from older population using face-to-face interview in Dhaka, Bangladesh. The prevalence of older adults aged above 60 was considered to calculate the study's sample size [25]. People diagnosed with severe mental and physical handicaps were not included in the study. The prevalence of older people aged above 60 was 8.4% [31]. With a 95% confidence interval and 5% margin of error, the sample size was calculated using the following formula:

(1) $$\text{Sample Size}=\frac{Z^{2}\times p\left(1-p\right)}{e^{2}}\times DEff\times NR$$

Where, Z-score for 95% confidence interval = 1.96; prevalence (p) = 0.084, margin of error (e) = 0.05; design effect (DEff) = 2.0 for sampling variation; non-response rate (NR) = 2%. The proposed sample size was 260. However, we collected 200 samples (60% for women and 40% for men) with a response rate of 91.9%. Systematic random sampling was applied for data collection due to resource constraints and simplicity in execution. Both men and women data collectors were recruited to address gender sensitivity and reduce potential reporting bias.

#### 2.1.2 Secondary data sources

##### 2.1.2.1 Demographic and Health Survey

National Institute of Population Research and Training (NIPORT) of the Ministry of Health and Family Welfare conducted the BDHS 2011, where Mitra and Associates, Bangladesh, implemented the survey. ICF International provided technical assistance as a part of its international Demographic and Health Surveys (DHS) Programme. An interview was conducted if the respondent provided their verbal consent in response to being read out an informed consent statement by the interviewer. The 2011 BDHS is the sixth DHS undertaken in Bangladesh. The sample for the BDHS 2011 is nationally representative, and it covers the entire population, and detailed methodology will be found elsewhere in study [25]. The total household was 17141, with members of 78909. We restricted the analysis only to information on the older population to address the research objective.

##### 2.1.2.2 Population and Housing Census

The practice of census taking in the Indian subcontinent, now Bangladesh, was started in 1872. After that, another census was conducted in 1881. Then, the census was conducted with the decennial periodicity and maintained (except in 1971 because of the liberation war). The first-ever census took place in 1974 in Bangladesh after her emergence as a newly independent nation in 1971. After that, Bangladesh went back to the decennial periodicity and held the second, third, fourth, and fifth censuses in 1981, 1991, 2001, and 2011. Details methodology of the census is available in the methodology section of the final report published by the BBS [29].

##### 2.1.2.3 Health and Morbidity Status Survey

Conducting various demographic and socio-economic surveys, BBS has developed an Integrated Multi-Purpose Sample (IMPS) design based on Population and Housing Census 2011. The Health and Morbidity Status Survey-2014 (HMSS-14) has been conducted throughout the country using the IMPS design of BBS, and detailed methodology can be found in the final report of HMSS [27]. The HMSS-14 was conducted among 37500 households, where 20025 were from the rural areas and 17475 from the urban areas. The total surveyed sample was 163057, where the weighted population above aged 60 years was 11193.

##### 2.1.2.4 Household Income and Expenditure Survey

HIES 2010 used a two-stage stratified random sampling technique based on the sampling frame of the Population and Housing Census 2001. The detailed methodology of HIES is available in the methodology part of the published report [30]. HIES collected data from 12240 households where 7840 from the rural area and 4400 from the urban area. The weighted and unweighted sample of the older adults was 4,189 and 10,978, respectively; 51% were men, and 49% were women.

### 2.2 Measures

#### 2.2.1 Age at marriage and life expectancy

First of all, we used mean age at marriage for both men and women. Estimation of age at marriage was made from census 2011. Gender difference in the mean age of marriage was calculated using the t-test. Secondly, we estimated life expectancy using age-specific population and age-specific death, which were taken from the census. Using age-specific population and death, we calculated the central death rate (age-specific death rate) [1]. Using the following equation, we derived the probability of dying ($q_{x}$).

(2) $$q_{x}=\frac{n.m_{x}}{1+\left(n-a_{x}\right)m_{x}}$$

Where n indicates the length of the age group and a_x_ indicates the mean number of person-years lived in the interval between age x and x + n by those dying in the interval. We used a_x_ equals 0.5 except for age 0 and 1 year. For age 0 and 1, we used 0.3 and 0.7, respectively, as a_x_. Using the probability of dying, we estimated life expectancy [1].

#### 2.2.2 Estimation of disability and disability-free life expectancy

BBS used six disability-related questions developed and adapted by the Washington Group [32] based on the World Health Organization's (WHO) the International Classification of Functioning, Disability, and Health (ICF) framework for conceptualising disability [33]. Six questions covered six functional domains of health: vision, hearing, walking and climbing, remembering and concentrating, self-care, and speaking and communicating. To assess and identify disabilities in the above six functional domains of health, the presence of any of six functional limitations were considered as disabled [20, 34]. After calculating the age-specific disability rate, using the Sullivan Method [35], we estimated disability-free life expectancy (DFLE) as follows:

(3) $$DFLE_{x}=\sum \frac{Lx*Dx}{lx}$$

*DFLE*_*x*_ is the disability-free life expectancy of a given age; *l*_*x*_ refers to the number of survivors at age x; *L*_*x*_ refers to the person-years lived for the age interval x, and *D*_*x*_ refers to the prevalence of disability-free for the age interval x.

#### 2.2.3 Estimation of morbidity and morbidity-free life expectancy

We estimated the prevalence of morbidity, comorbidity (two morbidities), and multimorbidity (more than two morbidities) using the morbidity information from HIES. Morbidity-free life expectancy (MFLE) was calculated by the same formula used for DFLE [35]:

(4) $$MFLE_{x}=\sum \frac{Lx*Mx}{lx}$$

*MFLE*_*x*_ is the morbidity-free life expectancy of a given age; *l*_*x*_ refers to the number of survivors at age x; *L*_*x*_ refers to the person-years lived for the age interval x, and *M*_*x*_ refers to the prevalence of morbidity-free for the age interval x.

#### 2.2.4 Estimation of independency, employment status, income, and health care utilisation

To estimate functional ability and independence, we derived the ability to work daily activities (for example- dressing, eating, washing, toileting) from HIES 2010 using activities of daily living (ADL). If a man or woman was currently unmarried or separated or divorced, or widow was considered alone. From the BDHS and HIES, we estimated employment and earning status. We considered purchasing health care from any facility as utilisation.

#### 2.2.5 Measuring depression and loneliness

The Geriatric Depression Scale-GDS (short version) was used to measure depression with a 15-item questionnaire [36]. The GDS questions are presented in Table 1, and a higher score reflects a greater depression. Generally, the index value higher than five is suggestive of depression, and a value equal to or higher than 10 is almost always indicative of depression [36]. The Cronbach alpha (α = 0.89) of these 15 items showed an excellent internal consistency. We also measured the older people's loneliness using the **De Jong Gierveld loneliness scale**, which comprises of 6-item questions (Table 1) [37]. The Cronbach alpha (α = 0.83) of these 6 items proved a good internal consistency. Similar to GDS, a higher value indicates more loneliness.

**Table 1:** Geriatric Depression Scale (GDS) short version and De Jong Gierveld loneliness scale.

Questions for depression	Yes	No
Are you satisfied with your life?	0	1
Have you released many of your activities and interests?	1	0
Do you think that your life is empty?	1	0
Do you often get bored?	1	0
Are you in a good state of mind most of the time?	0	1
Are you afraid that something terrible is going to happen to you?	1	0
Do you feel joyful most of the time?	0	1
Do you often feel helpless?	1	0
Do you prefer to stay at home, instead of going out and doing new things?	1	0
Do you sense that you have more difficulties with memory than others?	1	0
Do you think it is beautiful to be alive now?	0	1
Do you feel valueless the way you are now?	1	0
Do you feel full of energy?	0	1
Do you think that your current situation is hopeless?	1	0
Do you feel that most people are better off than you?	1	0

**Questions for loneliness**	**Yes**	**No**
Do you experience a general sense of emptiness?	1	0
Are there plenty of people you can rely on when you have problems?	0	1
Are there many people you can trust entirely?	0	1
Do you miss having people around?	1	0
Are there enough people you feel close?	0	1
Do you often feel rejected?	1	0

#### 2.2.6 Measuring active ageing index

The active ageing index (AAI) of the European Union (EU) scale shows the extent to which older people's potentials are realised [38]. The value of AAI indicates how older people are participating in society and economy and live healthy, secure, and independent life. All individual indicators from each domain (employment domain, independent, healthy and secure living domain, participation in the social domain, and capacity and enabling environment for active ageing domain), as shown in Table 2, were measured on the same scale, ranging from 0 (least positive result in terms of active ageing) to 100. In this way, by adding all indicators, we measured active ageing, and higher values indicated better outcomes in active ageing [38]. Data of all indicators were not available in Bangladesh, and therefore, we estimated it from the primary research. Finally, the AAI was calculated using weights for indicators and domains provided in Table 2, prepared based on UNECE and European Commission [38].

**Table 2:** Domains, Indicators, and Weights of Active Ageing Index (EU scale).

Domains	Domain weight within overall index	Indicators	Indicator weight within domain	Data Source
Employment	35	Employment rate 55–59 (%)	25	BDHS
		Employment rate 60–64 (%)	25	BDHS
		Employment rate 65–69 (%)	25	BDHS
		Employment rate 70–74 (%)	25	BDHS
		**Total**	**100**	
Participation in society	35	Voluntary activities (%)	25	Primary
		Care to children, grandchildren (%)	25	Primary
		Care to older adults (%)	30	Primary
		Political participation (%)	20	Primary
		**Total**	**100**	
Independent, healthy and secure living	10	Physical exercise (%)	10	HMSS
		Access to health and dental care (%)	20	HMSS
		Independent living (%)	20	BDHS
		Relative median income (%)	10	HIES
		No poverty risk (%)	10	HIES
		No material deprivation (%)	10	Primary
		Physical safety (%)	10	Primary
		Lifelong learning (%)	10	Primary
		**Total**	**100**	
Capacity and enabling environment for active ageing	20	Remaining life expectancy of 50 at 55 (%)	33	Calculated
		Share of healthy life expectancy at 55 (%)	23	Calculated
		Mental well-being (%)	17	HMSS
		Use of ICT (%)	7	HIES
		Social connectedness (%)	13	Primary
		Educational attainment (%)	7	BDHS
		**Total**	**100**	
**Total**	**100**			

**Figure 1:**
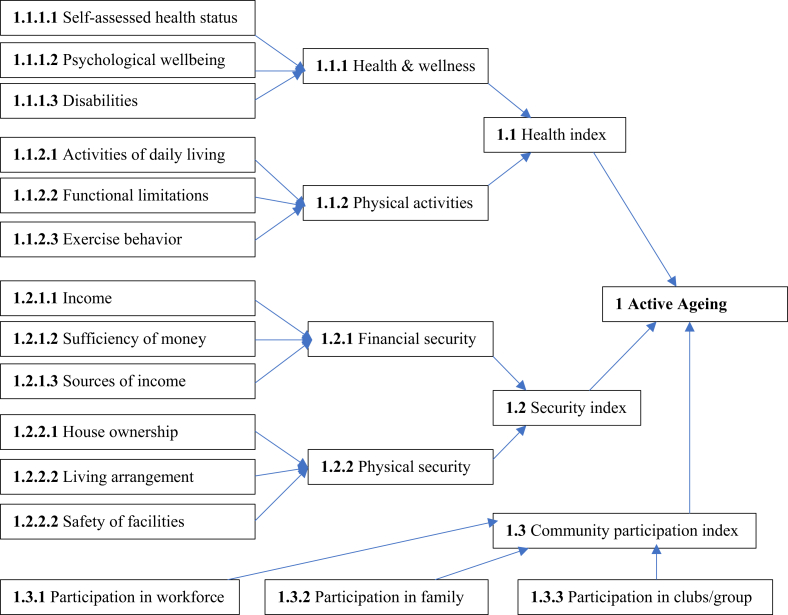
Active Ageing Index: WHO scale [39].

According to the WHO definition, active ageing has three components: health, community participation, and security (Figure 1). These three components have a total of 15 indicators: six indicators for health (three indicators for health and wellness, and three indicators for physical activities), three indicators for community participation, and six indicators for security (three indicators for financial security and three indicators for physical security) [39]. The actual score is calculated by summing the positive responses of the respondents in favour of their activeness [40]. Then we created an index for each dimension following the Human Development Index (HDI) constructed by the United Nations Development Programme [41]. Based on the UNDP criteria for levels of human development, also used by other researchers [40], we classified each index into three levels as follows: (a) low when an index value less than 0.5, (b) medium when an index value between 0.5 and 0.79, and (c) high when an index value equal to 0.8 and 1.

#### 2.2.7 Measuring quality of life: WHOQOL

**Figure 2:**
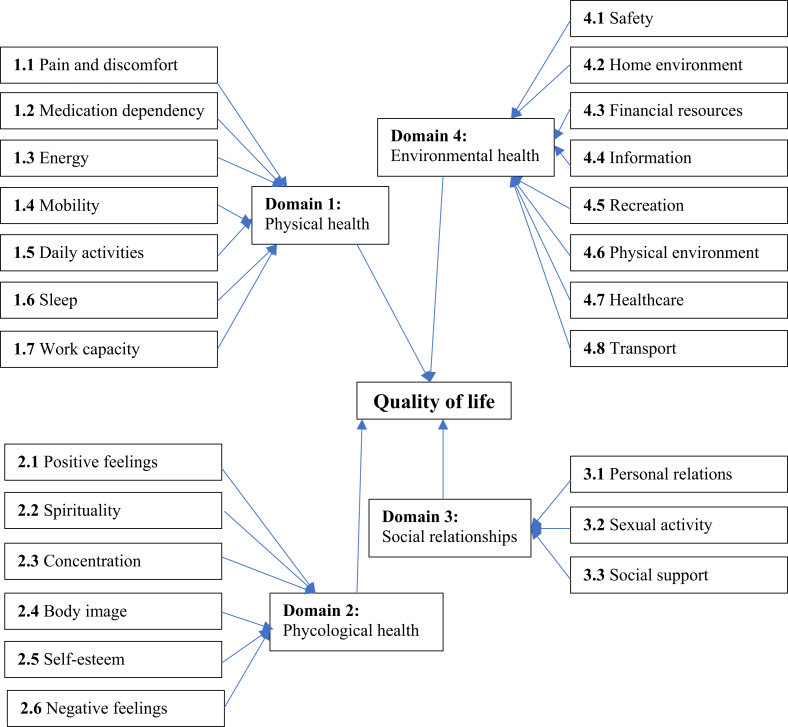
World Health Organization Quality of Life Instrument-Brief Version (WHOQOL-BREF [42].

We measured the quality of life (QoL) with the help of the validated World Health Organization Quality of Life Instrument-Brief Version (WHOQOL-BREF) [42], a culturally universal measure. It contains four domains: physical, psychological, social relations, and environmental (Figure 2). We followed WHO guidelines, calculated the QoL for the older population in Bangladesh, and detailed methodology would be found elsewhere in report [42]. The Cronbach alpha of these 24 items was 0.84, which showed good internal consistency. Furthermore, the higher index value offers a higher quality of life.

### 2.3 Analytical plan

We calculated mean age at marriage, life expectancy, disability, and multiple disabilities, disability-free life expectancy, morbidity, comorbidity, and multimorbidity, and morbidity-free life expectancy by gender. The independence status (functional ability), employment status, earning status, depression and loneliness status, and healthcare utilisation status was also calculated. We calculated the active ageing index and quality of life index as well. We employed the t-test, the Z-test (for life expectancy and healthy life expectancy only), and the Chi-square tests to examine the significance of the difference of indicators between men and women for understanding the Failures of Success (of higher longevity).

## 3 Ethical statements and data permission

This study did not need any ethical approval for secondary sources as they were de-identifiable. The NIPORT was responsible for BDHS, and it took ethical approval for the survey from Bangladesh Medical Research Council, and the data set was available at https://dhsprogram.com/data. The instructions were strictly followed for using the data of BDHS. BBS was responsible for the Census, HIES, and HMSS, and we took permission for using data. BBS collects data using verbal consent from the respondents. Data of BBS is available upon request (http://www.bbs.gov.bd/). However, institutional permission was taken from the department of population sciences, the University of Dhaka, for primary research. Most of the studies take verbal consent in Bangladesh due to the cultural sensitivity of written consent [25]. Moreover, more than 70% of the older population do not have any formal education (cannot read and write) [10]. In this regard, primary data were collected with verbal consent from the respondent using the following binary question: “do you agree to participate in this study after understating the aims and objectives of this research?” Participation in this research was voluntary, and there was no incentive for the respondents.

## 4 Findings

### 4.1 Mean age at marriage and life expectancy at birth

Table 3 presents the mean age at marriage and life expectancy at birth in Bangladesh. The mean age at marriage was 25.2 and 19.4 years for men and women, respectively, while life expectancy at birth was 67.7 and 70.7 years. Men had 5.8 years of higher mean age at marriage but three years lower life expectancy at birth than women. As a result, women would have to live, on average, 8.8 years alone. The mean age at marriage and the average life expectancy were higher in urban areas than rural areas.

**Table 3:** Mean age at marriage and life expectancy at birth in Bangladesh.

Domain	Indicators	Man	Woman	Difference	P-value
Urban	Mean Age at Marriage^a^	26.6	20.4	6.2	<.001
	Life Expectancy at Birth^b^	67.9	70.8	-2.9	<.001
Rural	Mean Age at Marriage^a^	24.7	19.0	5.7	<.001
	Life Expectancy at Birth^b^	66.6	70.5	-3.9	<.001
Total	Mean Age at Marriage^a^	25.2	19.4	5.8	<.001
	Life Expectancy at Birth^b^	67.6	70.7	-3.1	<.001

**Note:** a indicates that p-values were calculated using t-test; b refers to that p was computed using the z test. **Source**: Author's calculation and analysis of sample census 2011.

### 4.2 Disability, morbidity, healthy life expectancy, and healthcare utilisation

#### 4.2.1 Disability and morbidity-free life expectancy and functional ability

Table 4 illustrates the disability, morbidity, and health care utilisation of the older population in Bangladesh. A single disability among men was 39.3%, which was 7.8% points higher among women (47.1%). Like a single disability, the presence of multiple disabilities (more than one disability simultaneously) was also higher among women than that of men (23.9% vs 16.1%). We observed higher disability among older women, hence is not surprising that women would have a lower disability-free life expectancy. The study also found that 62.6% of older women suffered from any morbidity, which was 3.2% lower among older men. The comorbidity (at least two morbidities) was also higher among women than men. Also, older women suffered more from multimorbidity (more than two morbidities) than men. Morbidity-free life expectancy was higher among women as well. Older women would live six months more with morbidity than men. The difference in most of the indicators between men and women was statistically significant.

**Table 4:** Disability and morbidity-free life expectancy and functional ability among the older population by gender in Bangladesh.

Condition for older population	Man (95% CI)	Woman (95% CI)	Difference (95% CI)
**Disability**			
At least one disability (%)^**1**^	39.3 (38.0, 40.8)	47.1 (45.8, 48.5)	-7.8 (-7.9, -7.7)∗∗∗
More than one disability (%)^**1**^	16.1 (15.2, 17.1)	23.9 (22.8, 25.1)	-7.8 (-7.8, -7.8)∗∗∗
Disability-free life expectancy (year)^e^	6.9 (6.0, 7.4)	5.9 (5.2, 6.3)	1.0 (-0.1, 1.9)

**Morbidity**^**1**^			
No morbidity (%)^**1**^	37.4 (36.9, 39.4)	34.2 (33.5, 36.1)	3.2 (3.1, 3.3) ∗∗∗
At least one morbidity (%)^**1**^	62.6 (60.5, 63.1)	65.8 (64.0, 65.5)	-3.2 (-3.3, -3.1)∗∗∗
At least two morbidity (%)^**1**^	35.6 (34.4, 36.9)	38.4 (37.5, 40.1)	-2.8 (-2.9, -2.7)∗∗∗
More than two morbidities (%)^**1**^	18.5 (17.0, 19.0)	23.3 (22.8, 25.0)	-4.8 (-5.0, -4.6)∗∗∗
Morbidity-free life expectancy (year)^e^	4.2 (3.5, 4.9)	3.6 (3.1, 4.1)	0.6 (0.3, 0.9)

**Health Care Utilisation**			
Proportion of health care utilisation (%)^**2**^	61.4 (60.1, 62.7))	38.6 (37.5, 39.7)	22.8 (22.2, 23.4)∗∗∗
Mean cost of utilisation (BDT)^**1**^	436.3 (179, 694)	183.6 (156, 211)	252.7 (-6, 512) ∗

**Marital status**			
Living with spouse (%)^**1**^	91.1 (90.3, 91.8)	31.5 (30.2, 32.7)	65.4 (64.1, 65.7)∗∗∗
Living alone (%)^**1**^	8.9 (8.5, 9.3)	68.5 (67.3, 69.7)	-77.4 (79.7, -75.1)∗∗∗

**Employment and Earning**			
Employment status (%)^**3**^	57.8 (57.1, 58.5)	3.0 (2.9, 3.1)	54.8 (53.2, 56.4)∗∗∗
Earning status (%)^**1**^	59.8 (58.5, 61.1)	4.7 (4.1, 5.3)	40.6 (39.5, 41.7)∗∗∗

**Independency (Functional Ability)**			
Ability to work daily activities (%)^**1**^	60.7 (59.4, 62.0)	52.9 (51.5, 54.2)	7.8 (7.7, 7.9)∗∗∗

∗∗∗p ≤ 0.001, ∗∗p ≤ 0.01, ∗p ≤ 0.05; **1 =**HIES-2010; **2** = HMSS-2014; **3** = BDHS-2011; **e** refers to analysis using census-2011 and HIES-2010; 95% CI = 95% Confidence Interval.

#### 4.2.2 Proportion of disability and morbidity free life expectancy among older adults

The proportion of disability and morbidity free life expectancy among the older persons are depicted in Table 5. Life expectancy at birth and life expectancy in the older population was significantly higher among women than men. We observed that though women had a higher life expectancy, they were expected to live lower disability-free life expectancy. Women were expected to live 12.6 years with a disability, while it was 9.1 years for men. At the older age, men and women would have 5.3 and 6.6 years of life expectancy with a disability, respectively. At birth, the proportion of disability-free life expectancy was 86.5% and 82.2% for men and women, while in the older population, it dropped to 56% and 47%, respectively. The proportion of disability was higher among the more ageing population and women.

**Table 5:** Proportion of disability and morbidity free life expectancy among older population.

	Men	Women	Difference
**Life expectancy**			
Life expectancy at birth (year)	67.6	70.7	-3.1∗∗∗
Life expectancy at older age (year)	12.2	12.5	-0.3

**Disability and disability-free life expectancy**			
Disability-free life expectancy at birth (year)	58.5	58.1	0.4
Disability-free life expectancy at older age (year)	6.9	5.9	1.0
Life with disability at birth (year)	9.1	12.6	-3.5∗∗∗
Life with disability at older age (year)	5.3	6.6	-1.3
Proportion of disability-free life expectancy at birth (%)	86.5	82.2	4.3∗∗∗
Proportion of disability-free life expectancy at older age (%)	55.5	47.2	8.3∗∗∗

**Morbidity and morbidity-free life expectancy**			
Morbidity-free life expectancy at birth (year)	49.0	47.5	1.5∗
Morbidity-free life expectancy at older age (year)	4.2	3.6	0.6
Life with morbidity at birth (year)	18.6	23.2	-4.6∗∗∗
Life with morbidity at older age (year)	8.0	8.9	-0.9
Proportion of morbidity-free life expectancy at birth (%)	72.4	67.2	5.2∗∗∗
Proportion of morbidity-free life expectancy at older age (%)	33.2	28.8	4.4∗∗∗

**Source**: Author's calculation based on census 2011 report and HIES 2010 data. ∗∗∗p ≤ 0.001, ∗∗p ≤ 0.01, ∗p ≤ 0.05.

The difference between men's and women's morbidity-free life expectancy was 1.5 years at birth and six months at the older age. After birth, men and women were expected to live 18.6 and 23.2 years, respectively, with any morbidity, and the proportion of morbidity-free life expectancy was 72.4% and 67.2%, respectively. The difference was nearly similar to the older age (33.2% vs 28.8%). With being older, people were suffering more from both disability and morbidity.

#### 4.2.3 Regional variation in life expectancy and disability-free life expectancy by gender

Regional variations in life expectancy and disability-free life expectancy by gender are provided in Table 6. Irrespective of Bangladesh's regions (division), the difference between life expectancy and disability-free life expectancy was higher among women than men, indicating women had lower healthy life expectancy.

**Table 6:** Regional variation in life expectancy and disability-free life expectancy by gender.

Domain	Men						Women					
	LE_0_	DFLE_0_	D_0-0_	LE_65_	DFLE_65_	D_65-65_	LE_0_	DFLE_0_	D_0-0_	LE_65_	DFLE_65_	D_65-65_
Bangladesh	67.6	58.5	12.6	16.3	9.8	6.5	70.7	58.1	12.6	16.9	8.9	8.1
Barisal	67.6	59.7	7.9	16.3	10.2	6.1	70.7	58.8	11.9	16.9	7.9	9.1
Chattagram	63.8	57.3	6.5	16.4	11.2	5.1	70.0	60.3	9.6	17.0	10.1	6.9
Dhaka	68.0	58.8	9.2	16.2	9.6	6.6	71.6	58.9	12.7	16.9	8.7	8.2
Khulna	69.6	59.4	10.2	16.5	9.2	7.3	71.6	58.6	13.0	16.8	9.4	7.5
Rajshahi	70.3	56.8	13.5	16.5	7.8	8.7	71.7	54.5	17.2	17.0	7.2	9.8
Rangpur	69.1	58.6	10.5	16.4	9.6	6.8	68.6	53.9	14.8	17.0	7.9	9.2
Sylhet	67.2	59.9	7.3	16.0	10.6	5.4	70.5	61.4	9.0	17.0	10.9	6.0

**Note:** LE_0_ = life expectancy at birth; DFLE_0_ = Disability-free life expectancy at birth; D_0-0_ = Difference between life expectancy and disability-free life expectancy at birth; LE_65_ = life expectancy at age 65; DFLE_65_ = Disability-free life expectancy at age 65; D_65-65_ = Difference between life expectancy and disability-free life expectancy at age 65.

**Source**: Author's calculation based on census 2011 report and HIES 2010 data.

#### 4.3 Employment and earning

Table 4 also presents the marital status, employment status, and functional ability of the older persons. Both employment and earning status was very much lower for women. Only 3% of women were employed (both paid and unpaid), but only 2.4% were earning while about 58% of men were earning. Like earning status, around 61% of men could work daily actives, which was about 53% for women.

### 4.4 Loneliness and depression

Like all other indicators, loneliness was even higher among women (μ = 1.8; 95% CI = 1.5, 2.1) than men (μ = 1.4; 95% CI = 1.1, 1.7; p = 0.049). More than 91% of men lived with their spouses, while it was only 31.5% for women (Table 5). In contrast, 68.5% of women lived alone while only 8.5% of men. It was not surprising that women (μ = 7.3, 95% CI = 6.8 to 7.8) suffered more from depression than men (μ = 6.1, 95% CI = 5.7 to 6.6, p = 0.010).

### 4.5 Active ageing index

**Figure 3:**
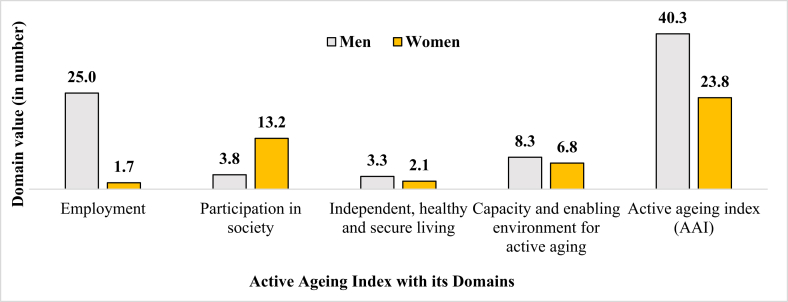
The Value of Active Ageing Index (EU scale) and Its Domain by Gender in Bangladesh. **Source**: Author's calculation based on BDHS 2011, census 2011 report, HMSS 2014, HIES 2010 data, and primary data.

Figure 3 shows the value of the active ageing index (AAI) for the EU scale. The score of the employment domain was 25.0 for men and 1.7 for women. Men were more involved in employment than women. However, the value of participation in society was higher for women. Women had lower scores in the independent, healthy, and secure living domain; and capacity and enabling environment for the active ageing domain. Finally, the AAI value showed that older men participated more in the economy and society and lived independent, healthy, and secure lives than women.

Moreover, the value of AAI as per the WHO scale was also lower among older women. The health index value was 0.67 (95% CI = 0.65, 0.69) for women and 0.70 (95% CI = 0.67, 0.73) for men. Men were more involved in community participation (0.42 vs 0.55) and had higher security (0.56 vs 0.72) than women. Overall, the value of AAI was 0.55 (55%; 95% CI = 51%, 59%) and 0.66 (66%; 95% CI = 61%, 71%) for older women and men which was moderate level as UNDP criteria.

### 4.6 Quality of life

**Figure 4:**
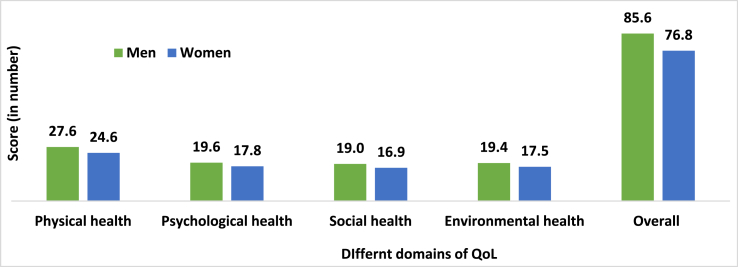
The Value of Quality of Life (QoL) Index and Its Domain by Gender in Bangladesh. **Source**: Author's calculation based on primary data.

The score of quality of life measures is presented in Figure 4. Quality of life of the older men was significantly higher in all domains, including physical health (27.6 vs 24.6), psychological health (19.6 vs 17.8), social health (19.0 vs 16.9), and environmental health (19.4 vs 17.5) than women. Moreover, the overall QoL scores were around 10 points (85.6 vs 76.8) higher for men than women (p < 0.001).

## 5 Discussions

The study addressed whether the higher life expectancy at birth was a gain or burden (Failures of Success) for older women in Bangladesh. The findings of this study have both similarities and dissimilarities with existing studies. Our study revealed that life expectancy was 67.6 years for men and 70.7 years for women. Simultaneously, the sample vital registration system (SVRS) report showed that life expectancy at birth was 67.9 years and 70.3 years, respectively [10]. Singh-Manoux et al. found little evidence for gender differences in the association between morbidity and mortality; they concluded that gender differences were not a valid explanation for the gender paradox [43]. Nevertheless, they also observed excess mortality among men and some excess morbidity among women. Excess mortality led to lower life expectancy in men. For instance, higher life expectancy was due to reduced maternal mortality, women's inherent biological superiority to men and the reflections of behavioural differences between them [7, 8, 44] as well as a higher accidental death among men. All existing studies showed that life expectancy at birth was higher in urban areas than rural [10, 18]. This study also revealed a similar scenario. For instance, life expectancy was inversely related to levels of rurality, and the gaps of life expectancy between rural and urban populations were widening [45]. However, unplanned urbanisation and increasing urban slums in Bangladesh would reverse the situation.

The findings of our study showed that the prevalence of disability among the older population was 43.1%, but existing studies reported that the prevalence was less than 15% in Bangladesh [46, 47]. Considering the broader dimensions of disability from the Washington group would be a probable explanation in this regard [33]. In line with the existing study, we found higher disability for women [46, 47, 48]. Women reported more disabilities, but most were non-fatal [7, 48]. As a result, men could be involved in more daily activities than women. Since the prevalence of disability and morbidity was higher among women than men, it was not surprising that life expectancy with morbidity and disability was also higher. Gender differences in disease prevalence can explain women's higher morbidity but lower mortality [7]. Moreover, women experience some biological advantages regarding reproduction, reducing their risk of cardiovascular disease before menopause [7].

Our study also showed that the prevalence of depression was disproportionately higher in older women than men, which was similar to existing studies as well [49, 50]. The higher burden of depression might be attributable to a greater susceptibility to persistent [49, 50] and heritability of depression [51]. Once women depressed more persistently, there would be a lower probability of death [49]. Therefore, despite higher depression, women had lower mortality. Moreover, men with depression sometimes hide their emotions and often seem irritable, angry, or aggressive [52]. Depression is often unrecognised in male-dominated industries among men [53]. As a result, the level of depression became lower among men, while many women expressed sadness resulting in higher depression.

Loneliness was also higher among women than men, which may cause emotional distress and is often linked to various health problems. Life changes, including widowhood, are associated with increased vulnerability to loneliness for older women [54]. Regarding self-labelling, women admit more frequently than men being lonely because the negative consequences of admitting loneliness are less for women [55]. Around 68.5% of older women were widowed in Bangladesh, and they were expected to live longer alone. However, levels of loneliness are similar for men and women across the lifespan [56].

Like many developing countries, especially Asian countries, employment and earning status were lower among women than men [7, 38]. Due to patriarchy [26], men were more likely to have paid employment than women. Our study also demonstrated that the value of the employment domain of the active ageing index was higher among men than women, which is also similar to existing literature [38]. Men were more involved in employment than women. In Bangladesh, most women engaged in the informal economy and unpaid labour in the household. As a result, women's work is not considered as employment. In contrast, women scored higher in the participation domain of AAI (EU scale) than men since they took care of more of their children or grandchildren or older family members than men. Household chores are often synonymous with the ‘responsibilities of women’ in the patriarchal society. As a result, women scored less in the independent, healthy, and secure living domain and capacity and enabling environment for the active ageing of AAI. Overall, the value of AAI was higher for men than women, and existing studies also showed a similar relationship [23, 38]. For instance, the AAI using the WHO scale was also lower for older women, and Tareqe et al. also showed similar findings in the context of Rajshahi, Bangladesh [23]. Overall, the quality of life, an essential component of individuals' general well-being for older adults, was also lower among older women than men and the existing studies also showed similar findings [57, 58, 59]. The lower quality of life for older women in Bangladesh was due to lower physical, mental, social, and environmental well-being.

Gender differences in disease prevalence, manifestation, and treatment are deeply rooted in the genetic differences between men and women [60]. For instance, heart disease is the leading cause of death accounted for 24.2% and 21.8% of all deaths for men and women, respectively, in the USA. Women were suffering from any diseases more than men; however, they were less likely to receive evidence-based treatment in developing countries [7, 60]. Similarly, healthcare utilisation was also lower among older women in Bangladesh.

Globally, the discourse on gender and health frequently asserts that older women are more vulnerable to social, economic and health disadvantages than men [7]. Most societies generally observe a nearly universal pattern of longer life expectancy among women. However, in itself, longer life conveys relatively little about the quality of life and burden of non-fatal disease during the extended years [7]. In this regard, the notion, ‘*women get sicker, but men die quicker*’ is often treated as a fact. Social explanations, such as stratification, social structure effect, and gender-specific normative behaviours and physiological differences were the determinants in this regard. Bird and Rieker developed a framework of ‘constrained choice’, which describes how decisions made and actions were taken at the levels of family, work, community and government shape men's and women's opportunities to pursue health [14]. Constrained choices at each level of society contribute to health differences, enhancing or limiting individual health behaviours and choices. These decisions can reduce individual agency for women but opposite for men.

Finally, there was no compression of morbidity in recent times in Bangladesh as older adults were suffering more chronic diseases, which is also similar to another study [17]. However, this study overall found that there were the ***Failures of Success*** for older adults, especially for older women in Bangladesh, as increasing life expectancies led them to extra years of chronic illness, economic insolvency, more anxiety and depression, and increasing misery. In this regard, Gruenberg also showed similar findings and expressed that we should search for preventable causes of the chronic illnesses which have been extending. He stressed that we would not move forward in enhancing health until we prevent non-fatal chronic illness [15].

## 6 Conclusions and implications

The well-known empirical evidence that women live longer than men but, paradoxically, they report higher rates of morbidity and disability has also been observed in Bangladesh. Despite higher longevity, women are more prone to morbidity, disability, unemployment, depression, and loneliness which is the ***Failures of Success***. There are sharp differences and variations in regional life expectancy. Thus, a single (similar) policy and programme is not appropriate for all regions. However, all kinds of variations and differences in life expectancy indicate apparent inequalities and gaps. Therefore, population (segmented) specific intervention should be taken to reduce the inequalities and gaps. Women have a higher life expectancy but a lower quality of life than men. The findings would be helpful for intervention and policy implications in this circumstance. The government and policymakers may intervene for improving the quality of life, especially for women. Without considering the quality of life, sustainable development goals, mainly goal number three of good health and well-being, cannot be achieved. Also, we cannot ensure healthy ageing without the quality of life. Finally, sex and gender are the first and foremost modifiers of disease pathophysiology, presentation, and response to treatment. Gender influences on the behaviour of the family, society, and community can be considered a social and psychological modifier of morbidity and mortality. In this regard, sex and gender and their inherent differences should inform decision making to promote gender equity in health.

This study has some compelling strengths, as it was the first attempt to address whether the higher life expectancy at birth was a gain or burden (Failures of Success) for older women in Bangladesh. We analysed large nationwide samples that gave enough information and increased its acceptability to another setting. Despite such strengths, the study has some limitations. The study was based on self-reported data, and self-reported data is vulnerable to social desirability and recall bias. We calculated life expectancy using the age-specific death rate, but the cause-specific death rate may produce more accurate results to compare the competing risk of men and women. We considered complex sampling for BDHS data, but we could not consider it for BBS data due to the unavailability of cluster variables in the data. We collected primary data from 200 older populations (for missing variables to measure all aspects of quality of life) which was small; regional variations were not considered. We could not estimate the quality of life indicators for the cohorts of women who lived less than men, for example, before 2000, and compare with the results from this study to see whether any changes in quality of life has accompanied the changing life expectancy. All the active ageing domains (EU scale) are not applicable in Bangladesh's context. However, we used to measure the situation of the older population, not for scale validation. The disparities between men and women might exist because of measurement/sampling error. Finally, QoL is a more qualitative aspect rather than quantitative. As a result, a qualitative study can produce more in-depth insights into the gain and burden of higher life expectancy.

## Declarations

### Author contribution statement

Md. Zakiul Alam: Conceived and designed the experiments; Performed the experiments; Analyzed and interpreted the data; Contributed reagents, materials, analysis tools or data; Wrote the paper.

### Funding statement

This research did not receive any specific grant from funding agencies in the public, commercial, or not-for-profit sectors.

### Data availability statement

The authors do not have permission to share data.

### Declaration of interests statement

The authors declare no conflict of interest.

### Additional information

No additional information is available for this paper.
